# A Large Exophytic Tumor of the Cervix Causing Vaginal Bleeding in Pregnancy: A Case Report

**DOI:** 10.7759/cureus.35747

**Published:** 2023-03-04

**Authors:** Nektaria Zagorianakou, Alexandros Katrachouras, Nadia Almousa, Chara Skentou, George Makrydimas

**Affiliations:** 1 Cytology, University of Ioannina, Ioannina, GRC; 2 Obstetrics and Gynecology, University Hospital of Ioannina, Ioannina, GRC; 3 Obstetrics and Gynecology, University of Ioannina, Ioannina, GRC

**Keywords:** cervical neoplasia, giant condyloma, vaginal bleeding, pregnancy, exophytic tumor of the cervix

## Abstract

Vaginal bleeding in the second and third trimesters of pregnancy is usually due to placental causes, namely placental abruption and placenta previa. Other causes include uterine rupture, vasa previa, and hematologic disorders. However, benign or malignant lesions of the vagina and the cervix may also cause vaginal bleeding or spotting. Although cervical cancer in pregnancy is rare, about 8% of pregnant women have an abnormal Pap smear and 3% of the total cervical cancers are diagnosed during pregnancy. We report a case of a 20-week pregnant woman who presented with vaginal bleeding; a visual inspection revealed a large exophytic lesion of the cervix. The Pap smear demonstrated a low-grade squamous intraepithelial lesion (LSIL) related to human papillomavirus (HPV) infection. The differential diagnosis based on the findings of the colposcopy included invasive cervical carcinoma, warty lesions, and perishable lesion. A cesarean section and the removal of the cervical tumor were scheduled and carried out as planned at 37 weeks of gestation. The histologic examination showed extensive lesions of low-grade squamous intraepithelial cervical neoplasia (LSIL/CIN1). Despite the fact that exophytic tumors of the cervix are extremely rare, in women presenting with vaginal bleeding or spotting during the second or third trimester of pregnancy, the ultrasound scan must be followed by a visual inspection of the vagina and the cervix.

## Introduction

Vaginal bleeding beyond the first trimester of pregnancy is far less common compared to the first trimester, and it is most frequently caused by placental abruption and placenta previa [1]. Other less common causes include uterine rupture, vasa previa, and hematologic disorders [1]. However, benign or malignant lesions of the vagina and the cervix may also cause vaginal bleeding or spotting. Therefore, in women presenting with vaginal bleeding or spotting during the second or third trimester of pregnancy, an ultrasound scan is performed to ensure fetal viability and establish the location of the placenta, followed by a visual inspection of the vagina and the cervix.

Local causes of bleeding from the cervix comprise infections, injuries, intraepithelial neoplasia, and cancer. With an incidence rate of 1.2 per 10,000 pregnancies in the US, cervical cancer is one of the most common malignancies discovered during pregnancy [2,3], while up to 5% of pregnant women have been reported to have an abnormal Pap smear, which is comparable to that in nonpregnant women [3,4-6].

We discuss a case of a pregnant woman who presented at 20 weeks of gestation with vaginal spotting; during speculum examination of the cervix, a large exophytic tumor was identified, arising from the endocervix.

## Case presentation

A 24-year-old pregnant woman at 20 weeks of gestation, with no history of previous pregnancies, presented to the outpatient department of our clinic due to spotting after sexual intercourse. Her medical history was unremarkable. During this pregnancy, she had routine antenatal care and normal blood tests, and the first-trimester scan at 12 weeks of gestation demonstrated a normal fetus, low risk for trisomies, and development of preeclampsia or fetal growth restriction.

**Figure 1:**
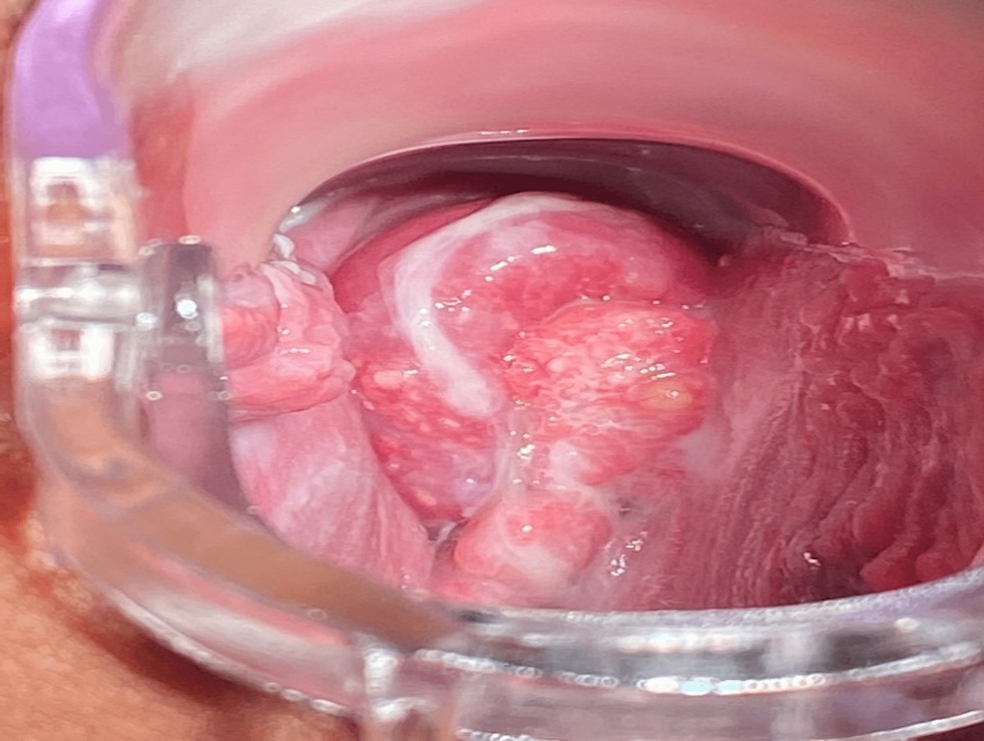
The gross image of the cervical exophytic lesion

An ultrasound scan was performed on admission, which confirmed fetal viability with no obvious fetal malformations and the placenta was high anterior. Visual inspection of the vagina and the cervix identified a large, hyperemic tumor with spotty foci of bleeding arising from the cervix (Figure 1).

A Pap smear was obtained from the cervix and the tumor. The patient was referred for a colposcopy, which was arranged a week later.

The Pap smear demonstrated a low-grade squamous intraepithelial lesion (LSIL) related to human papillomavirus (HPV) infection. The cytological findings reported the presence of koilocytes, and mature squamous cells with clearly recognizable nuclear and cytoplasmic alterations. They were characterized by perinuclear cavitation, binucleation, nuclear hyperchromasia, and nuclear enlargement. The cytological hallmark of an LSIL is the koilocyte (hollow cell).

**Figure 2:**
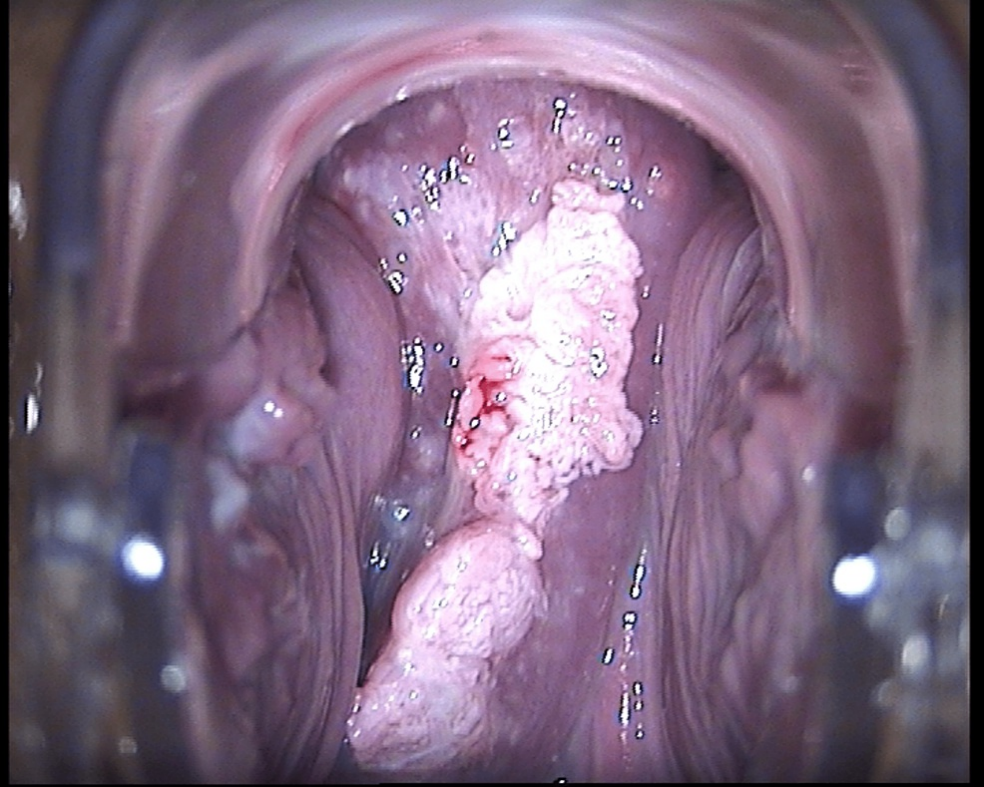
Colposcopic image of the cervical exophytic lesion

Colposcopy was carried out by an experienced clinician. The differential diagnosis included (1) invasive cervical carcinoma, (2) warty lesions, and (3) decidual reaction (Figure 2).

The option of surgical removal of this lesion was discussed with the patient and her partner. After intensive counseling and considering the risks for the pregnancy associated with the operation, the couple opted to avoid the surgical removal of the tumor at this stage and proceed with the pregnancy up to 36-37 weeks. A cesarean section was scheduled and carried out as planned at 37 weeks of gestation. A healthy female newborn was delivered, with a fetal weight of 2880 gr. Immediately after the cesarean section, the cervical tumor was removed and sent for histological examination.

The histology results were as follows: macroscopic examination recognized three irregularly shaped white-brown web pieces of total dimensions of 2 x 1.8 x 0.4 cm. Microscopic examination of the presented material identified endocervical mucosa with extensive squamous metaplasia of the mainly overlying and less of the glandular epithelium, which focally assumed the character of premature (immature) metaplasia. The overlapping multilayered squamous epithelium showed capable hyperplasia, papillomatosis, and focal hyperkeratosis with parakeratosis. The papillary structures dived into the dermis and had a smooth circumference. Basal/delinquent cell hyperplasia was evident and was accompanied by an increased nucleocytoplasmic ratio and nuclear motility. The immunohistochemical examination revealed that the protein p16 was focally expressed and the proliferation index Ki67 mainly in the basal and parabasal layers of the epithelium. Finally, cystic-arranged endocervical glands were observed.

The final histology report documented lesions of low-grade squamous intraepithelial cervical neoplasia (LSIL/CIN1). There was no clear evidence to suggest invasive squamous cell carcinoma. The patient was followed up with a colposcopy two months after delivery, which was unremarkable. Afterward, the patient had four more Pap smears every six months, all of which were normal.

## Discussion

This case primarily indicates that although vaginal bleeding in pregnancy is mainly caused by placental abnormalities, it can also result from local causes, and, secondly, that visual inspection of the vagina and the cervix followed by the appropriate diagnostic procedure is of critical importance. Malignant and possibly life-threatening conditions should be diagnosed as early as possible in order to improve maternal and neonatal outcomes. In this case, vaginal bleeding was caused by a large exophytic tumor arising from the cervix. The appearance of the tumor was considered suspicious and therefore a Pap smear was taken immediately, followed by a colposcopy.

Cervical cancer in pregnancy is rare; 1-3% of cervical cancers are diagnosed during pregnancy, or conversely, 0.05% of all pregnancies are complicated by cervical cancer [2]. Interestingly, Zemlickis et al. have reported that cervical cancer in pregnancy is more likely to be diagnosed in earlier stages and this is in accordance with the results of other studies reporting that pregnant women are more likely to be diagnosed in the International Federation of Gynecology and Obstetrics (FIGO) Stage I compared to nonpregnant women and the most likely explanation for this difference is the more intensive medical care received by pregnant women [6-8]. Most of these women are young and apparently healthy and have never seen a medical doctor before. Therefore, routine medical tests are performed for the first time and several medical conditions, including cervical cancer, may be diagnosed by these tests [6].

Although it has been reported that the prevalence of HPV infection in pregnancy is similar to that in nonpregnant women of the same age, other studies have shown a significant increase in pregnancy [9,10]. This was attributed to hormonal changes and possibly to immunological factors, such as immunosuppression in pregnancy, that could facilitate the reactivation of the virus and the consequent cervical dysplasia and cancer [11]. Therefore, screening in pregnancy using cervical cytology is important and all pregnant women who had not had a recent Pap smear should be advised to perform one. When cytology suggests SIL during pregnancy, the same diagnostic procedure is followed as in nonpregnant women. However, due to the physiological changes during pregnancy, such as increased stromal edema, vascularization, and cervical volume, the evaluation of colposcopy and cytology often becomes difficult [5].

In our case, the Pap smear demonstrated LSIL related to HPV infection. HPV infection may present with varying alterations in the morphology of the squamous and glandular epithelium. Usually, these lesions are flat, but in some cases, they are exophytic with acanthosis and hyperkeratosis. Although these tumors are more frequently found in the vulva, perineum, and perianal skin, in rare cases, they can arise from the cervix [12,13]. It is unusual for patients who have LSIL on the Papanicolaou test to have an invasive lesion when they are biopsied. A retrospective study reported that none of the patients with low-grade dysplasia was found to have invasive cancer [14]. According to another study, 62% of patients with antepartum LSIL cytology experienced disease remission after delivery, 32% had persistent LSIL on subsequent Pap tests, and only 6% experienced HSIL progression. None of them had invasive cancer [4]. On the other hand, only 1% of the patients with HSIL cytology will be found to have invasive cancer [15].

The differential diagnosis from the findings of the colposcopy included invasive cervical carcinoma, warty lesions, and decidual reaction. Immediate surgical removal or diagnostic biopsy of the lesion could cause bleeding, abortion, or preterm birth, while delaying diagnosis and treatment to minimize the risks for the neonate could put the mother at risk, in case of a malignancy. Detailed counseling of the parents regarding the therapeutical options and the associated risks for the mother and the fetus was performed by the obstetrician, the oncologist, and a psychologist. Based mainly on the opinion of the oncologist who had performed the colposcopy that the chances of an invasive lesion were rather small, the parents opted to avoid immediate excision of the tumor and instead perform a cesarean section beyond 36 weeks of gestation.

A written consent form for the publication of this case report was obtained from the patient.

## Conclusions

Benign or malignant lesions of the vagina and the cervix may cause vaginal bleeding or spotting. Therefore, in women presenting with these symptoms during the second or third trimester of pregnancy, an ultrasound scan is performed to ensure fetal viability and establish the location of the placenta, followed by a visual inspection of the vagina and the cervix. Exophytic tumors of the cervix are extremely rare, and when diagnosed, the possibility of malignancy must be ruled out. The routine diagnostic procedure includes cytology and/or HPV typing and colposcopy. The decision on further diagnostic tests (biopsy) and the appropriate timing for surgical treatment should be taken after considering the risks for the mother and the pregnancy.
